# Re-initiation of CT colonography services during the COVID-19 pandemic: Preliminary evaluation of safety

**DOI:** 10.1259/bjr.20201316

**Published:** 2021-04-09

**Authors:** David Peprah, Andrew Plumb, Alison Corr, Janice Muckian, Kathryn Smith, Antoni Sergot, Jia Ying Kuah, James Stephenson

**Affiliations:** 1 University College London Hospitals NHS Foundation Trust, London, UK; 2 St Mark’s Hospital NHS Trust, Harrow, United Kingdom; 3 Imperial College Healthcare NHS Trust, London, United Kingdom; 4 Gastrointestinal Imaging Group, University Hospitals Leicester NHS Trust, Leicester, United Kingdom

## Abstract

**Objective:**

The COVID-19 pandemic has led to cancellation and deferral of many cancer investigations, including CT colonography (CTC). In May 2020, BSGAR and SCoR issued guidelines outlining steps for conduct of CTC in the early recovery phase. We evaluated the implementation of these in four English hospital trusts.

**Methods:**

Ethical permission was not required for this multicentre service evaluation. We identified patients undergoing CTC over a 2-month period from May to July 2020 at four Trusts. We recorded demographics, scan indications, colonic findings, and incidental lung base changes compatible with COVID-19. A subset of patients were contacted via telephone to document new symptoms 2 weeks following their scan. Staff were contacted to determine if any acquired COVID-19 during the period.

**Results:**

224 patients (118 male, 52.7%) were scanned during the period. In 55 patients (24.6%), CTC showed a ≥6 mm polyp. 33 of 224 (14.7%) scans showed incidental lung base changes felt unrelated to COVID-19, and only one patient had changes indeterminate for COVID-19; no classic COVID-19 pulmonary changes were found. Of 169 patients with telephone follow-up, none reported any new symptoms of COVID-19 (cough, fever, anosmia, ageusia) within 14 days of CTC. None of the 86 staff contacted developed COVID-19.

**Conclusion:**

We found no cases of patients or staff acquiring COVID-19 infection following CTC; and no evidence of significant asymptomatic COVID-19 patients attending for CTC appointments based on lung base changes.

**Advances in knowledge:**

Our findings suggest that current practice is unlikely to contribute significantly to spread of SARS-nCOV2. Cancer and significant polyp detection rates were high, underlining the importance of maintaining service provision.

## Introduction

The current COVID-19 pandemic has affected all aspects of world affairs in an extraordinary and unexpected manner. Since the virus was first identified in Wuhan, China in December 2019, the number of cases has risen alarmingly. According to the WHO update in mid-September 2020, there have been 29.5 million cases, with 931,000 confirmed deaths spread across 216 countries.^1^ Inevitably, restrictions have been introduced in an attempt to reduce transmission. In the UK, this ultimately led to a “lockdown” during which residents were encouraged to “Stay Home, Protect the NHS, Save Lives”.

As part of “protecting the NHS”, a significant amount of elective and routine (but nonetheless necessary) work was either cancelled or deferred. Remote consultations have become the norm, and (at least initially) patients were encouraged to access medical advice via 111 rather than visiting GP practices.^2^ Accordingly, there has been significant disruption to service provision that has necessarily affected clinically urgent activity, including outpatient cancer investigations. This includes the 2 week-wait (2WW) referral pathway, which saw activity reductions of up to 80% during the pandemic peak in March and April.^3^ According to the National Endoscopy Database (NED), which captures a large proportion of endoscopic activity in England in near real-time, during April and May 2020, endoscopic activity was only 12% of pre-COVID levels.^4^ Such delayed diagnosis is predicted to lead to substantial increases in cancer deaths, ranging from 4.8 to 5.3% increase for lung cancer to a 15.3–16.6% increase for colorectal cancer (CRC), ultimately causing around 1500 extra deaths within 5 years for CRC alone.^3^

There has been understandable concern among members of the public about visiting hospitals during the pandemic. It is unlikely that these concerns will resolve rapidly without an effective and widely available vaccine against SARS-nCOV2, the causative agent of COVID-19. However, it is critical that patients who need investigation are able to access such tests both rapidly and safely. CT colonography (CTC) is an appealing alternative to colonoscopy for investigation of colorectal symptoms, since it is rapid, not currently designated as an aerosol-generating procedure, only requires intubation of the low rectum with a thin disposable catheter, and does not need the operator to stand close to the patient during the test, thus facilitating social distancing. The British Society of Gastrointestinal and Abdominal Radiology (BSGAR) and the Society and College of Radiographers (SCoR) issued guidelines for the conduct of CTC during the recovery phase of the COVID-19 pandemic,^5^ emphasizing the need to protect patients and staff from viral transmission.

Here we report the results of a multicentre service evaluation of the implementation of these guidelines at four separate CTC units in England.

## Methods

### Site and patient population

This was a prospective, multicentre service evaluation; ethical permission was not required. Patients who underwent CTC over a 2 month period from 11 May to 8 July 2020 were included in the study (*i.e.,* almost immediately after publication of the joint guidance on 5 May). The data were collected from four major hospital trusts in England – A, B, C and D. All sites were implementing the BSGAR-SCoR guidance for CTC. In brief, this required (a) droplet PPE precautions for staff, including a visor during rectal cannulation and insufflation, (b) pre-test symptom screening for patients and (c) routine cleaning of the CT scanner in between patients.

### Data collection

At each site, a local investigator identified patients undergoing CTC on a weekly basis using the hospital Radiology Information System (RIS) and/or Electronic Patient Record (EPR) and/or Picture Archiving and Communication System (PACS). All CTCs were reported via the normal clinical pathway. Using the CTC reports, the local investigator extracted; (a) patient demographics, (b) scan indication, (c) colonic findings, coded according to the English Bowel Cancer Screening Programme minimum dataset recommendations as Cx (inadequate study), C1 (normal, benign lesion, or 1 to 2 polyps of ≤5 mm), C2 (1 to 2 polyps, 6 to 9 mm), C3 (C3*a* = 3 to 4 polyps, 1 to 9 mm; C3*b* = 1 to 2 polyps, at least one polyp ≥10 mm; C3*c* = indeterminate stricture), C4 (C4*a* = 5 or more polyps, smaller than 10 mm; C4*b* = 3 or more polyps, at least one ≥10 mm) or C5 (C5*a* = colonic mass characteristic of malignancy; C5*b* = no tumour additional to colonoscopy findings), (d) the presence of lung base changes, and (e) whether or not these changes were compatible with SARS-nCOV2 infection.

Subsequently, at three of the four sites (A, B, C), patients were followed up via telephone at 14 days after their CTC, and were asked to report if they had developed a new fever, cough, or loss of taste or smell within 14 days of the CTC appointment; or received a diagnosis of COVID-19 since their CTC.

In addition, radiographic staff performing the CTCs at the various sites were surveyed and asked in retrospect to report if any had developed symptoms suggestive of SARS-nCOV2 infection over the study period.

Data were collated using Microsoft Excel (Redmond, USA) and IBM^®^ SPSS V.26.0.

## Results

**Figure 1.**
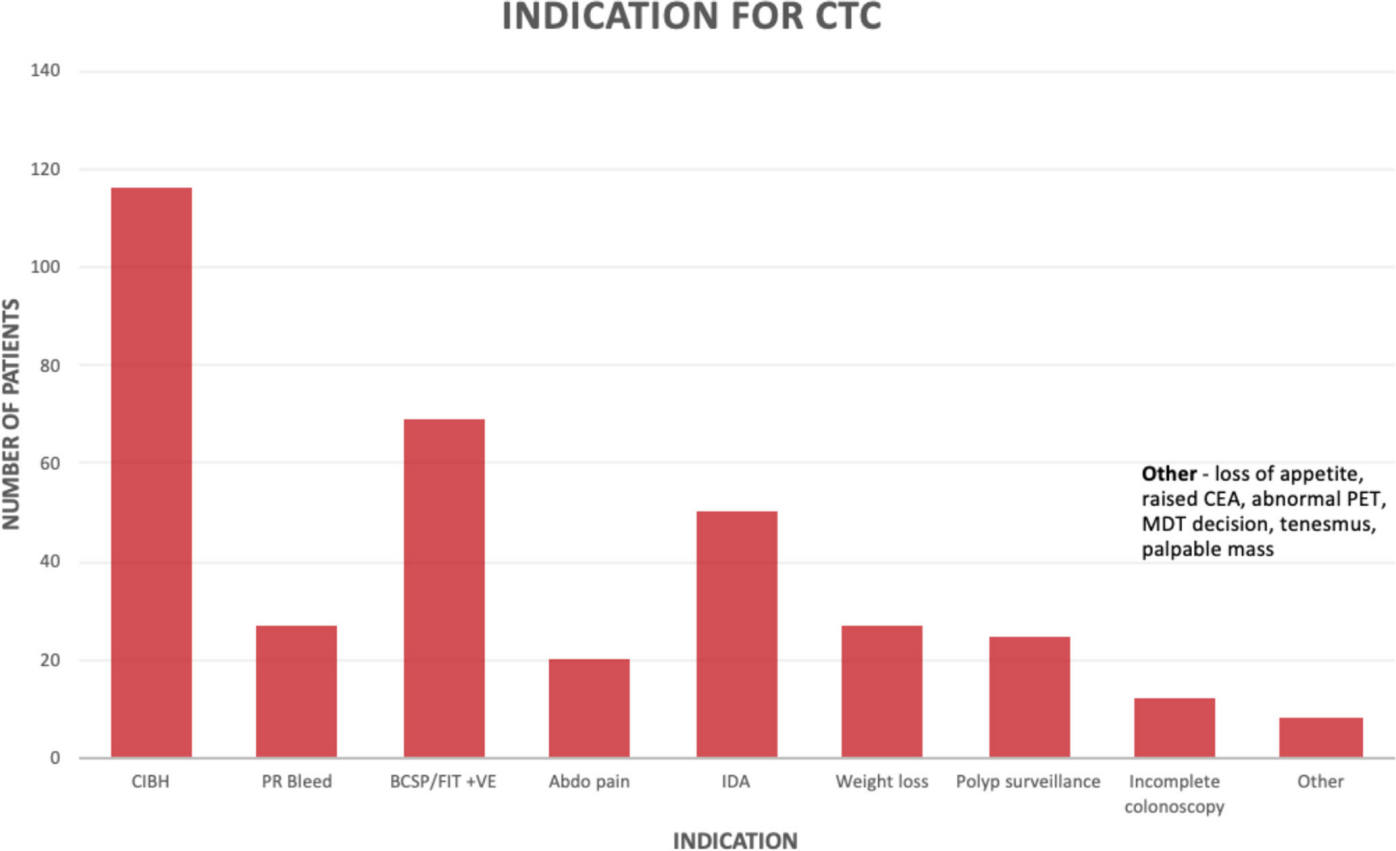
Indications for CTC.

During the 2-month period, data were available for 224 patients undergoing CTC, of whom 106 (47.3%) were male and 118 (52.7%) were female. The age ranged from 41 to 91 years, with a mean of 71.2 years. The commonest indications for CTC were: change in bowel habit (116/224; 32.8%), a positive faecal immunochemical test (69/224; 19.5%), iron deficiency anaemia (50/224; 14.1%), weight loss (27/224; 7.6%), bleeding per rectum (27/224; 7.6%), polyp surveillance (25/224; 7.1%), and abdominal pain (20/224; 5.6%; Figure 1).

The majority of CTCs were normal; 160/224 (71.4%) received a C1 code. Inadequate studies (Cx) were uncommon (9/224; 4%), meaning the remaining 55 studies (24.6%) showed at least one polyp of 6 mm or greater. In 25 cases (11.2%), a cancer or polyp of ≥10 mm was diagnosed. 11 patients (4.9%) were diagnosed with CRC (Table 1).

**Table 1.** Colonic findings at CTC. C1 (normal, benign lesion, or 1 to 2 polyps of ≤5 mm), C2 (1 to 2 polyps, 6 to 9 mm), C3 (C3*a* = 3 to 4 polyps, 1 to 9 mm; C3*b* = 1 to 2 polyps, at least one polyp ≥10 mm; C3*c* = indeterminate stricture), C4 (C4*a* = 5 or more polyps, smaller than 10 mm; C4*b* = 3 or more polyps, at least one ≥10 mm) or C5 (C5*a* = colonic mass characteristic of malignancy; C5*b* = no tumour additional to colonoscopy findings; Cx = inadequate study)

**Colonic Findings**	**Number of Scans**	**Percentage**
**C1**	160	71.4%
**C2**	19	8.5%
**C3a**	7	3.1%
**C3b**	10	4.5%
**C3c**	1	0.45%
**C4a**	3	1.3%
**C4b**	4	1.8%
**C5a**	10	4.5%
**C5b**	1	0.45%
**Cx**	9	4%

**Figure 2.**
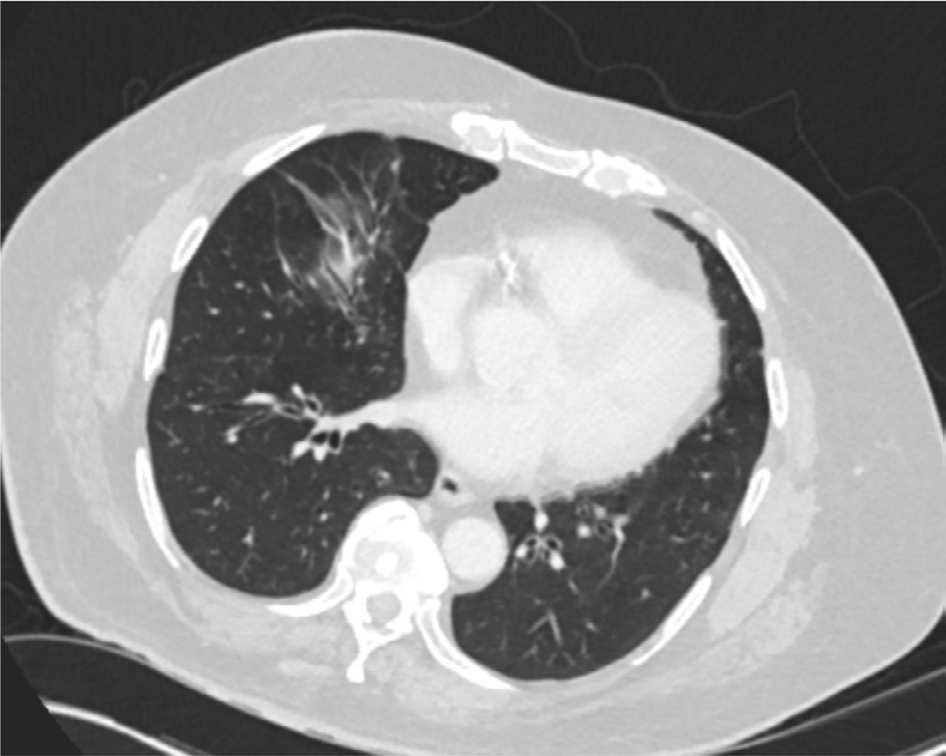
Middle lobe pulmonary changes in the single patient regarded as indeterminate for COVID-19 infection.

Regarding lung base changes, 190 of the scans (84.8%) revealed no lung base changes. 33 of 224 (14.7%) scans showed incidental lung base changes felt unrelated to COVID-19. Only 1 of 224 (0.4%) patients was reported to have lung base changes potentially compatible with COVID-19 infection (Figure 2). On subsequent review of the images, the features were felt indeterminate for COVID-19, and no such diagnosis was ultimately made in this asymptomatic patient.

169 (75.4%) of patients scanned were followed up via a telephone consultation. Of these, no patients reported any new symptoms within 14 days of CTC, that is, new cough, new fever, new anosmia or ageusia (loss of taste).

In total, 86 staff were contacted from the four different sites, none of whom developed symptoms suggestive of SARS-nCOV2 infection over the scanning period.

## Discussion

Our results provide encouraging preliminary data characterising the risk of COVID-19 in patients receiving CTC at four UK centres in the early post-peak (first wave) phase. The recent combined BSGAR-SCoR guidance for the conduct of CTC was designed to balance the competing risks to patients of possible exposure to healthcare-acquired infection (HAI) by SARS-nCOV2 *vs* that of delayed diagnosis. Moreover, the NHS has a duty to protecting its staff from acquiring COVID-19 as far as practical. Therefore, it is of considerable importance to establish that current recommended practice is indeed safe for both parties.

The time of infection by SARS-nCOV2 to the development of symptoms suggesting COVID-19 is under 14 days with cough, fever, anosmia and ageusia amongst the most frequent symptoms. Reassuringly, of the patients we contacted, none reported new symptoms suggestive of COVID-19 within this period. Although with relatively small numbers this cannot be regarded as proof that no such nosocomial transmission occurred, the upper bound of the 95% confidence limit for zero cases from 169 patients is 2.2%, meaning the risk is likely less than 1 in 50, and probably considerably smaller.

In terms of inadvertent exposure of staff to patients with undiagnosed SARS-nCOV2 infection, in the absence of routine pre-CTC viral swabbing of patients, we used a proxy measure, that is, lung base changes. Although this is clearly imperfect, particularly for asymptomatic disease, we found no changes of “classic” COVID-19 according to British Society of Thoracic Imaging criteria, and only 1 (0.45%) with changes compatible with COVID-19 infection. Moreover, on questioning radiology department staff who performed these CTC examinations, none were diagnosed with COVID-19 during the relevant service evaluation period. Recent data from 6208 outpatient endoscopy procedures from 18 centres showed a very low prevalence of unsuspected SARS-nCOV2 infection; 2611 patients had undergone pre-endoscopy naso-pharyngeal swabs, and only three tested positive.^6^ No cases of patient-to-staff or staff-to-patient transmission were documented when following UK guidance for conduct of endoscopy, although this differs from (and is generally more intensive than) that for CTC. Nonetheless, we find it reassuring that there is consistent evidence, albeit imperfect, that current practice is unlikely to be contributing significantly to spread of SARS-nCOV2.

We found a relatively high rate of colonic abnormalities (26.8% with a ≥ 6 mm polyp or cancer), substantially greater than that previously reported in prior UK series of CTC which ranged from 17 to 18.3%.^7–9^ This is likely to reflect the fact that only (clinically) higher-risk patients were being routinely investigated during the study period, including use of FIT triage. Given we found a 4.5% prevalence of cancer, 1.5 times above the 2WW target of 3%, this underlines the importance of prompt investigation, even in the era of SARS-nCOV2, if we are to avoid the harms of delayed diagnosis.

Clearly, there are limitations inherent to our findings. We used a cross-sectional design that only provides a snapshot over a specific period. Therefore, it may not accurately predict the risk as the pandemic evolves and community prevalence changes. Fluctuations in numbers of cases and risk of community transmission will inevitably affect the prevalence of asymptomatic disease that may lead to staff exposure to SARS-nCOV2. Our sample size was small, meaning rare events (such as COVID-19 transmission) may be missed. Although we used a range of centres, these may not reflect the wider UK. Individual sites or regions may wish to monitor their own situation to better tailor their services and precautions to local disease prevalence.

## Conclusion

In summary, we report early results from re-initiation of CTC services in the early post-COVID-19 recovery phase following the first wave. We found no cases of patient or staff infection after CTC, and little to no evidence of patients attending for CTC with asymptomatic COVID-19 as judged by lung base changes. Detection rates of cancer and significant polyps were substantially greater than most previous reports of CTC, emphasizing the need for ongoing investigation of high-risk patients.
